# Associations of patient-generated subjective global assessment (PG-SGA) and NUTRISCORE with survival in gastric cancer patients: timing matters, a retrospective cohort study

**DOI:** 10.1186/s12876-022-02515-3

**Published:** 2022-11-17

**Authors:** Jae Won Cho, Jiyoung Youn, Eun Mee Kim, Min-Gew Choi, Jung Eun Lee

**Affiliations:** 1 Department of Dietetic, Samsung Medical Center, 06351 Seoul, Korea; 2 Department of Food and Nutrition, Seoul National University, 1 Gwanak-ro, Gwanak-gu, 08826 Seoul, Korea; 3 Department of Surgery, Samsung Medical Center, Sungkyunkwan University School of Medicine, 06351 Seoul, Korea; 4 Research Institute of Human Ecology, Seoul National University, 08826 Seoul, Korea

**Keywords:** Gastric cancer, Gastrectomy, Malnutrition, PG-SGA, NUTRISCORE, Survival

## Abstract

**Background:**

The timing of nutritional assessment may be important to treat cancer patients and predict their prognosis. This study examined whether Patient-Generated Subjective Global Assessment (PG-SGA) and NUTRISCORE scores were associated with survival among gastric cancer patients who underwent surgery and chemotherapy and whether the timing of the assessment after surgery mattered.

**Methods:**

A total of 952 gastric cancer patients (622 men and 330 women) were included in this retrospective cohort study. The PG-SGA and NUTRISCORE scores were calculated at 1 month (*n* = 952), 2 months (*n* = 657), and 3 months (*n* = 294) after surgery. Cox proportional hazards model was used to calculate the hazard ratios (HRs) and 95% confidence intervals (CIs).

**Results:**

The PG-SGA scores assessed at 1 month after gastrectomy were not associated with survival. However, high PG-SGA scores at 2 months after gastrectomy (median = 65 days) were associated with an increased risk of mortality; the HR (95% CI) was 2.26 (1.22–4.21) for 9–11 vs. ≤ 5 of PG-SGA scores. When we included patients who received all three consecutive consultations, HR (95% CI) was 2.56 (1.02–6.42) for ≥ 9 (malnutrition) vs. ≤ 8 of PG-SGA scores assessed at 3 months after surgery (median days = 98 days). Likewise, high NUTRISCORE scores assessed at the 3-month follow-up were associated with higher mortality; the HR (95% CI) was 3.84 (1.18–12.55) for ≥ 7 vs. ≤ 4 of NUTRISCORE scores.

**Conclusion:**

Malnutrition assessed with the PG-SGA and NUTRISCORE at 2 to 3 months after gastrectomy was associated with poor survival among gastric cancer patients. Our findings suggest that the timing of the nutritional evaluation may be important in identifying and treating malnutrition related to gastric cancer prognosis.

**Supplementary Information:**

The online version contains supplementary material available at 10.1186/s12876-022-02515-3.

## Background

Gastric cancer is the fifth leading cause of cancer death worldwide [1], and over half of gastric cancer cases occur in Asia [2]. Stomach cancer is the third most common cancer in Korea, accounting for 11.6% of all cancers in 2019 [3] and the death rate in 2020 is 9.1% of all cancer deaths, which is fourth [4].

The common curative treatment for gastric cancer is gastric resection surgery combined with chemotherapy or radiation therapy [5, 6]. Gastric cancer patients who underwent chemotherapy after surgery often exhibit weight loss and malnutrition, leading to toxicity and shorter survival [7, 8] by promoting tumor invasion and deteriorating immune competence and tolerance to treatment [9].

The Patient-Generated Subjective Global Assessment (PG-SGA), consisting of weight change, dietary intake change, nutrition impact symptoms during the past two weeks, activities and function, and physical examination, has been widely used for cancer patients as a nutritional evaluation tool [10]. The NUTRISCORE is a relatively new nutritional screening tool that determines the risk of malnutrition for oncological outpatients based on weight loss, reduced food intake, and the types of cancer and anticancer therapy [11].

Several studies examined the association between PG-SGA scores and mortality among cancer patients. A prospective cohort study from Brazilian women with gynecologic tumors suggested that malnourished patients with a PG-SGA score above 10 measured during the first 24h of hospitalization were 30.7 times higher risk of mortality [12]. A retrospective study of patients diagnosed with multiple myeloma (MM) reported that a higher PG-SGA score measured at the beginning of first-line chemotherapy was associated with reduced survival [13]. Another retrospective study of patients diagnosed with advanced lung cancer in northern China showed that malnourished status (PG-SGA scores ≥ 9) assessed at admission was associated with a 1.9 times higher risk of mortality [14]. Several studies demonstrated evaluating nutritional status using the NUTRISCORE in cancer patients [15, 16]. However, to our knowledge, no studies have investigated the association of the NUTRISCORE with gastric cancer mortality.

Because treating malnutrition is essential to improving gastric cancer prognosis, identifying appropriate nutritional assessment methods and assessing nutritional status are crucial. Also, the timing of the nutritional evaluation may be important because of variation in nutritional status across time since the surgery or treatment. However, only a limited number of studies conducted repeated assessments of nutritional status before or after gastrectomy using the nutrition risk index (NRI) or prognostic nutritional index (PNI) [17, 18]. No studies have reported multiple assessments of the PG-SGA or NUTRISCORE for gastric cancer patients.

This study examined whether the PG-SGA and NUTRISCORE scores and the timing of their assessment were associated with survival among gastric cancer patients who underwent postoperative chemotherapy.

## Methods

### Study population

**Fig. 1:**
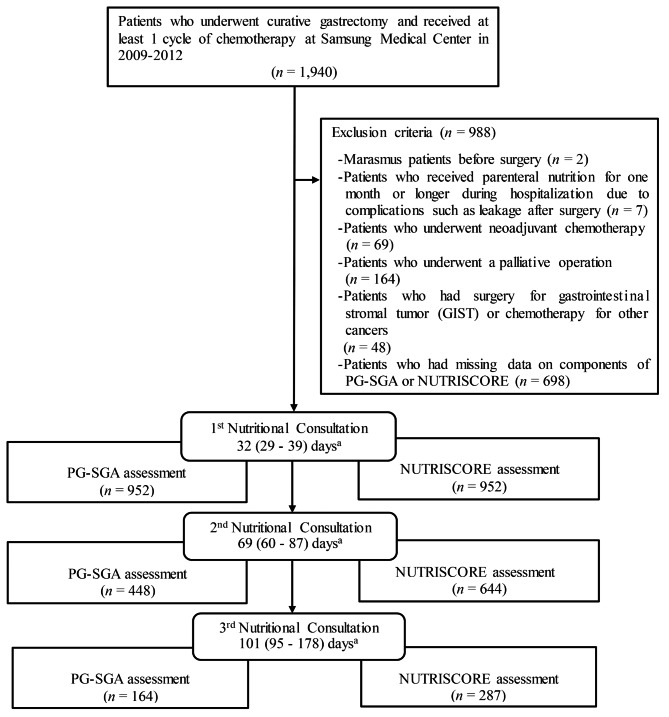
Flow diagram of study population inclusion *PG-SGA* patient generated-subjective global assessment ^a^Median (IQR) days since surgery among patients whose PG-SGA or NUTRISCORE assessment was available

This study was a retrospective cohort study of gastric cancer patients whose medical health institute data was linked with the death certificate database of the National Statistical Office in Korea. A total of 1,940 patients who underwent curative gastrectomy and received at least 1 cycle of chemotherapy at Samsung Medical Center between January 2009 and December 2012 were retrospectively identified by electronic medical records. We sequentially excluded the following patients: (1) malnutrition patients with marasmus identified by the International classification of Disease (ICD)-9 before surgery (*n* = 2); (2) patients who had been hospitalized for one month or more and received parenteral nutrition during hospitalization due to leakage after surgery (*n* = 7); (3) patients who underwent neoadjuvant chemotherapy (*n* = 69); (4) patients who underwent a palliative operation (*n* = 164); (5) patients who underwent surgery for gastrointestinal stromal tumor (GIST) or chemotherapy for other cancers (*n* = 48); or (6) patients who had missing data on any of the components of the PG-SGA or NUTRISCORE (*n* = 698) (Fig.1). As a result, a total of 952 patients (622 men and 330 women) aged from 24 to 82 years were analyzed in this study. This study was approved by the institutional review board of Samsung Medical Center, Korea (IRB No. 2017-11-025).

### Assessments of clinical and nutritional factors

Information on the date of birth, sex, the types of operation (subtotal gastrectomy (STG) or total gastrectomy (TG)), disease progression of gastric cancer (early gastric cancer (EGC) or advanced gastric cancer (AGC)) [19], method of anticancer therapy (chemotherapy (CTx) or combined chemoradiotherapy (CCRTx)), stage of gastric cancer, and postoperative hospital days were obtained from the medical records. The stage of gastric cancer was determined according to the eighth edition of the American Joint Committee on Cancer (AJCC) Tumor–Node–Metastasis (TNM) staging system [19]. Information on serum levels of albumin, hemoglobin, total lymphocyte count (TLC), and total cholesterol measured at one month after surgery was obtained through the medical record. Height and weight were directly measured by a digital height and weight scale at each visit. Body mass index (BMI) was calculated by dividing body weight (kg) by height squared (m)^2^. Information on weight loss, the number of meals and snacks, eating speed, and digestive symptoms were collected through face-to-face interviews by registered dietitians during nutritional consultation. Energy and protein intake was assessed by registered dietitians using a one-time 24h recall.

### Calculation of the PG-SGA and NUTRISCORE

We evaluated nutritional status of patients using PG-SGA prior to starting nutritional consultations. The scoring system of the PG-SGA includes patient-completed components (weight loss, food intake, clinical symptoms, activities, and function) and clinician-completed components (disease, metabolic demand, and physical examination), with higher scores indicating higher levels of malnutrition. The nutritional triage recommendations were as follows: 0–1, no intervention required at this time; 2–3, education required by a dietitian; 4–8, intervention required by a dietitian; and ≥ 9, intensive intervention required by a dietitian [20, 21]. The NUTRISCORE is a recently developed nutritional screening tool for oncology outpatients and consists of the patient’s answers (weight loss and reduction in food intake due to poor appetite) and medical data (cancer location and type of treatment). NUTRISCORE was calculated according to the manual based on the collected data [11]. The NUTRISCORE includes two scores for the upper gastrointestinal tract location and two scores for CCRTx, or one score for CTx according to the treatment method, considering the tumor site and the oncologic treatment [22]. The NUTRISCORE 5 or above indicates that the patient is at risk of malnutrition [11].

### Follow-up

Nutrition education and counseling were conducted one month after surgery, that is, at the beginning of chemotherapy. During the chemotherapy, if the patient wanted additional nutritional consultations at the hospital visit, 1 or 2 more sessions were provided (Fig.1). At one month after gastrectomy, nutritional education is provided for patients starting anticancer therapy, and additional nutritional consultation is available during outpatient clinic visits or anticancer therapy visits. After the nutritional evaluation, a clinical dietitian conducted nutritional intervention to solve each patient’s nutritional problem, and provided dietary advice during chemotherapy and a balanced diet after therapy. The interval between visits to get nutritional consultation is the basis of determining the three time points. Median days since surgery at first to third nutritional consultations were 32, 69, and 101 days. Deaths of participants until December 31, 2016, were ascertained using the Korea National Statistical Office (KNSO) database. The cause of death was coded and classified according to the Korean standard classification of diseases 7 (KCD7). Gastric cancer deaths were identified with code C16.

### Statistical analysis

We compared patients’ characteristics by calculating the mean and standard deviations for continuous variables and the number and percentage for categorical variables according to PG-SGA and NUTRISCORE scores. Hazard ratios (HRs) and 95% confidence intervals (CIs) were obtained by using the Cox proportional hazards model [23]. Person-years were calculated from the date of each consultation to the death date or the end of the follow-up (December 31, 2016), whichever came first. Main analysis was performed according to either quartile of the PG-SGA and NUTRISCORE scores or the cut-off point suggested for intensive intervention (9 points for PG-SGA and 5 points for NUTRISCORE). Because of the higher number of patients in the 5 and 6 scores of the NUTRISCORE, the sizes of the quartile groups were not equal. In the multivariate model, we adjusted for sex, age at surgery (years, continuous), types of operation (STG or TG), stages (stage I&II or stage III), weight loss (kg, continuous), body mass index (BMI, kg/m^2^, continuous), digestive symptoms (yes or no), and protein intake relative to requirement (1.2 g/kg (current body weight)/day) [24] (≥ 75% or < 75%). The proportional hazards assumption was examined using an interaction term between the main exposure and the log of the follow-up time, and no violations were found (all *P* > 0.05). For the test for trends, the median value of each quartile group was included in the model as a continuous variable. We examined the association in subgroup analyses of sex and stage. In a sensitivity analysis, we limited our analysis to patients who received all three nutritional consultations (145 patients with a PG-SGA score and 278 patients with a NUTRISCORE score). Also, PG-SGA or NUTRISCORE was divided into two groups based on the cut-off points of nutritional intervention (9 scores for PG-SGA [13] and 5 scores for NUTRISCORE [11]). All analyses were performed using SAS 9.4 (SAS Institute Inc., Cary, NC). All statistical tests were two-sided, and P values < 0.05 were considered statistically significant.

## Results

The median follow-up duration was 65.9 months (range 6.0-95.7 months). The mean age at the time of surgery was 55.3 ± 11.5 years, and 622 (65.3%) were men. The proportion of women was higher in the highest category of PG-SGA scores than in the other categories (Table1). Patients in the highest category of PG-SGA scores had higher levels of weight loss and a higher proportion of TG compared to those in lower scores. Lower intakes of energy and protein, and higher chance of postprandial digestive problems were observed in the highest category of PG-SGA scores compared to the lower categories. According to the NUTRISCORE, younger age, higher proportion of men, higher weight loss, higher stage of gastric cancer, a higher proportion of CCRTx, lower intakes of energy and protein, and less frequent meal eating were observed in patients of the highest category than in the lower categories. Serum levels of albumin, hemoglobin, total lymphocyte count, and total cholesterol did not vary according to PG-SGA and NUTRISCORE scores.

**Table 1:** Baseline characteristics according to PG-SGA or NUTRISCORE scores at first nutritional consultation

Characteristics^a^	PG-SGA				NUTRISCORE			
	**≤ 5**	**6–8**	**9–11**	**≥ 12**	**≤ 4**	**5**	**6**	**≥ 7**
N (%)	177 (18.6)	229 (24.1)	256 (26.9)	290 (30.5)	135 (14.2)	313 (32.9)	336 (35.3)	168 (17.7)
Median scores	4	7	10	14	4	5	6	7
Person-years	836.2	1182.3	1341.6	1498.8	635.4	1624.3	1757.0	842.3
Age at surgery (years)	55.5 ± 11.9	53.8 ± 11.6	55.8 ± 11.4	56.0 ± 11.0	58.0 ± 12.1	55.0 ± 11.6	55.4 ± 11.3	53.8 ± 10.8
Sex								
Men	125 (70.6)	167 (72.9)	171 (66.8)	159 (54.8)	78 (57.8)	190 (60.7)	226 (67.3)	128 (76.2)
Women	52 (29.4)	62 (27.1)	85 (33.2)	131 (45.2)	57 (42.2)	123 (39.3)	110 (32.7)	40 (23.8)
Body Mass Index (kg/m^2^)	22.0 ± 2.7	22.0 ± 2.7	21.7 ± 2.7	21.6 ± 2.6	21.4 ± 2.8	21.7 ± 2.5	21.8 ± 2.7	22.3 ± 2.9
Weight loss (kg)	4.0 ± 3.0	5.0 ± 2.5	5.4 ± 2.4	5.9 ± 3.4	2.2 ± 3.4	4.1 ± 1.8	5.9 ± 1.8	8.1 ± 2.7
TNM stage								
Stage I and II	98 (55.4)	129 (56.3)	150 (58.6)	151 (52.1)	79 (58.5)	183 (58.5)	191 (56.9)	75 (44.6)
Stage III	79 (44.6)	100 (43.7)	106 (41.4)	139 (47.9)	56 (41.5)	130 (41.5)	145 (43.2)	93 (55.4)
Type of operation								
STG	124 (70.1)	160 (69.9)	166 (64.8)	168 (57.9)	88 (65.2)	230 (73.5)	196 (58.3)	104 (61.9)
TG	53 (29.9)	69 (30.1)	90 (35.2)	122 (42.1)	47 (34.8)	83 (26.5)	140 (41.7)	64 (38.1)
Disease progression								
EGC	32 (18.1)	28 (12.2)	37 (14.5)	52 (17.9)	16 (11.9)	50 (16.0)	55 (16.4)	28 (16.7)
AGC	145 (81.9)	201 (87.8)	219 (85.6)	238 (82.1)	119 (88.2)	263 (84.0)	281 (83.6)	140 (83.3)
Anticancer therapy								
CTx only	99 (55.9)	112 (48.9)	138 (53.9)	149 (51.4)	128 (94.8)	184 (58.8)	165 (49.1)	21 (12.5)
CCRTx	78 (44.1)	117 (51.1)	118 (46.1)	141 (48.6)	7 (5.2)	129 (41.2)	171 (50.9)	147 (87.5)
Postoperative hospital stay (days)	11.6 ± 4.9	11.3 ± 3.1	11.3 ± 3.5	11.5 ± 4.4	11.2 ± 2.6	11.2 ± 3.6	11.5 ± 4.3	12.0 ± 4.7
Albumin (g/dL)	4.2 ± 0.3	4.2 ± 0.3	4.2 ± 0.3	4.2 ± 0.4	4.1 ± 0.3	4.2 ± 0.3	4.2 ± 0.4	4.2 ± 0.4
Hemoglobin (g/dL)	12.1 ± 1.6	12.3 ± 1.5	12.4 ± 1.4	12.2 ± 1.4	11.9 ± 1.6	12.2 ± 1.4	12.2 ± 1.4	12.6 ± 1.5
TLC (per mm^3^)	2095.4 ± 634.5	2042.4 ± 664.6	2030.8 ± 719.8	2100.1 ± 738.5	2089.1 ± 627.9	2058.9 ± 712.1	2048.9 ± 723.6	2099.0 ± 672.0
Total cholesterol (mg/dL)	167.0 ± 30.0	163.0 ± 27.5	171.6 ± 32.0	168.5 ± 33.4	169.3 ± 32.5	171.0 ± 32.4	164.3 ± 29.2	167.2 ± 31.0
Energy intake (kcal/day)	1561.5 ± 386.6	1444.5 ± 342.9	1288.1 ± 340.2	1146.6 ± 375.2	1626.9 ± 352.4	1432.0 ± 358.0	1233.0 ± 375.3	1114.8 ± 310.1
Energy intake relative to requirement (%)	81.7 ± 17.7	75.9 ± 16.7	68.6 ± 17.1	62.8 ± 19.0	89.7 ± 12.9	77.7 ± 16.7	64.7 ± 16.6	56.4 ± 13.9
Protein intake (g/day)^b^	61.1 ± 20.1	58.6 ± 19.1	51.7 ± 17.7	45.1 ± 18.2	64.8 ± 17.7	57.4 ± 19.7	48.7 ± 18.2	44.2 ± 17.2
Protein intake relative to requirement (%)^b^	81.3 ± 24.1	78.1 ± 23.5	70.3 ± 24.5	62.4 ± 24.0	92.2 ± 19.6	79.1 ± 24.4	64.7 ± 22.3	56.2 ± 20.0
Number of meals (times/day)^b^	3.4 ± 0.9	3.5 ± 1.0	3.5 ± 0.9	3.4 ± 0.9	3.7 ± 1.1	3.5 ± 1.0	3.4 ± 0.9	3.3 ± 0.8
Number of snacks (times/day)^b^	2.8 ± 0.8	2.7 ± 0.7	2.8 ± 0.7	2.7 ± 1.5	2.8 ± 0.8	2.7 ± 0.7	2.8 ± 1.4	2.6 ± 0.6
Eating speed (minutes)^b^	20.5 ± 6.2	20.2 ± 6.4	20.3 ± 6.1	20.1 ± 6.9	20.4 ± 6.3	20.5 ± 6.5	20.1 ± 6.6	20.0 ± 6.0
Postprandial digestive problems (yes, %)	124 (70.1)	164 (71.6)	180 (70.3)	241 (83.1)	90 (66.7)	228 (72.8)	254 (75.6)	137 (81.6)

*PG-SGA* patient generated-subjective global assessment, *STG* subtotal gastrectomy, *TG* total gastrectomy, *EGC* early gastric cancer, *AGC* advanced gastric cancer, *CTx* chemotherapy, *CCRTx* combined chemoradiotherapy, *TLC* total lymphocyte count

^a^Values were presented as mean ± standard deviation for continuous variables or number (%) for categorical variables

^b^ There were fewer patients due to a lack of information: protein intake and protein intake relative to requirement, 894; the number of meals, 951; the number of snacks, 931; eating speed, 650

We examined whether the PG-SGA and NUTRISCORE scores at each consultation time point (1, 2, and 3 months) were associated with mortality (Table2). The PG-SGA scores at the 1st consultation were not associated with mortality. Meanwhile, high PG-SGA scores at the 2nd consultation time (median days = 65 days after surgery) were associated with a high risk of mortality; the HR (95% CI) for PG-SGA scores 9–11 compared to ≤ 5 was 2.26 (1.22–4.21). When we adjusted for anticancer therapy in addition to covariates in Model 3, HR (95% CI) was 2.25 (1.21–4.18) for PG-SGA scores 9–11 compared to ≤ 5. This positive association, although not significant, tended to persist for the PG-SGA scores assessed at 3 months after surgery (median days = 98 days after surgery). We found an inverse association for the NUTRISCORE assessed at 1 month (median days = 32 days after surgery). However, there was no association for the NUTSCORE assessed at 2 months. For the NUTRISCORE assessed at 3 months, we found a 3.84 times higher risk of mortality for the NUTRISCORE scores ≥ 7 compared to ≤ 4 scores.

**Table 2:** Hazard ratios (HRs) and 95% confidence intervals (CIs) of total mortality according to PG-SGA and NUTRISCORE scores

By time point	PG-SGA					NUTRISCORE				
	**≤ 5**	**6–8**	**9–11**	**≥ 12**	**P for trend**	**≤ 4**	**5**	**6**	**≥ 7**	**P for trend**
1st nutritional consultation										
N (score means ± SD)	177 (3.6 ± 1.4)	229 (7.1 ± 0.8)	256 (10.0 ± 0.8)	290 (15.3 ± 3.2)		135 (3.9 ± 0.3)	313 (5.0 ± 0.0)	336 (6.0 ± 0.0)	168 (7.1 ± 0.3)	
No. of cases/person-years	50/836.2	52/1182.3	58/1341.6	73/1498.8		47/635.4	72/1624.3	72/1757.0	42/842.3	
Model 1^a^	1.00	0.76 (0.51–1.12)	0.73 (0.50–1.07)	0.84 (0.58–1.21)	0.515	1.00	0.63 (0.44–0.92)	0.57 (0.39–0.82)	0.70 (0.46–1.07)	0.082
Model 2^b^	1.00	0.75 (0.51–1.11)	0.71 (0.49–1.04)	0.75 (0.52–1.08)	0.184	1.00	0.64 (0.44–0.92)	0.51 (0.35–0.74)	0.56 (0.37–0.86)	0.004
Model 3^c^	1.00	0.79 (0.53–1.17)	0.79 (0.53–1.17)	0.84 (0.57–1.24)	0.531	1.00	0.66 (0.45–0.97)	0.55 (0.35–0.86)	0.62 (0.35–1.10)	0.076
2nd nutritional consultation										
N (score means ± SD)	161 (3.1 ± 1.3)	102 (7.0 ± 0.8)	76 (10.1 ± 0.9)	109 (15.1 ± 2.8)		288 (3.8 ± 0.4)	241 (5.0 ± 0.0)	99 (6.0 ± 0.0)	16 (7.4 ± 0.6)	
No. of cases/person-years	26/816.4	20/503.4	23/382.4	22/551.5		57/1484.3	55/1232.8	23/494.2	4/80.9	
Model 1^a^	1.00	1.30 (0.72–2.35)	2.00 (1.14–3.52)	1.37 (0.77–2.45)	0.156	1.00	1.20 (0.83–1.75)	1.29 (0.79–2.11)	1.46 (0.53–4.04)	0.200
Model 2^b^	1.00	1.09 (0.60–1.98)	1.96 (1.10–3.49)	1.12 (0.62–2.04)	0.450	1.00	1.01 (0.69–1.47)	0.93 (0.56–1.55)	0.82 (0.29–2.30)	0.711
Model 3^c^	1.00	1.15 (0.61–2.15)	2.26 (1.22–4.21)	1.24 (0.64–2.43)	0.331	1.00	1.03 (0.68–1.55)	1.04 (0.59–1.87)	1.04 (0.32–3.41)	0.881
3rd nutritional consultation										
N (score means ± SD)	32 (3.3 ± 1.2)	31 (7.1 ± 0.7)	32 (10.1 ± 0.9)	69 (15.3 ± 2.9)		87 (3.7 ± 0.4)	93 (5.0 ± 0.0)	86 (6.0 ± 0.0)	21 (7.0 ± 0.2)	
No. of cases/person-years	6/165.8	4/174.3	9/156.5	18/372.5		14/454.4	28/446.8	14/489.3	7/96.5	
Model 1^a^	1.00	0.63 (0.17–2.26)	1.76 (0.59–5.28)	1.45 (0.56–3.77)	0.234	1.00	2.02 (1.06–3.86)	0.98 (0.46–2.12)	2.11 (0.84–5.30)	0.516
Model 2^b^	1.00	0.63 (0.18–2.26)	1.45 (0.47–4.48)	1.50 (0.57–3.93)	0.216	1.00	1.44 (0.74–2.79)	0.71 (0.33–1.53)	1.57 (0.61–4.09)	0.784
Model 3^c^	1.00	0.68 (0.18–2.63)	1.61 (0.49–5.27)	1.88 (0.60–5.89)	0.124	1.00	1.82 (0.90–3.67)	1.24 (0.49–3.12)	3.84 (1.18–12.55)	0.127

*PG-SGA* patient generated-subjective global assessment

^a^Model 1 was adjusted for age at surgery and sex

^b^Model 2 was adjusted for types of operation (STG or TG) and stages (stage I and II or stage III) in addition to variables in model 1

^c^Model 3 was adjusted for weight loss (kg, continuous), body mass index (kg/m^2^, continuous), digestive symptoms (yes or no), and protein intake relative to requirement (≥ 75% or < 75%) at each nutritional consultation in additional to variables in model 2

When we limited the analysis to cause-specific survival of deaths from gastric cancer, we found similar associations to those observed in the analysis in which all deaths were included. There was an increased risk of mortality among gastric patients for PG-SGA scores of 9–11 assessed at 2 months compared to ≤ 5 scores: HR (95% CI) = 2.45 (1.23–4.86) (Table3).

**Table 3:** Hazard ratios (HRs) and 95% confidence intervals (CIs) of deaths from gastric cancer according to PG-SGA and NUTRISCORE scores

By time point	PG-SGA					NUTRISCORE				
	**≤ 5**	**6–8**	**9–11**	**≥ 12**	*P* **for trend**	**≤ 4**	**5**	**6**	**≥ 7**	*P* **for trend**
1st nutritional consultation										
N (score means ± SD)	177 (3.6 ± 1.4)	229 (7.1 ± 0.8)	256 (10.0 ± 0.8)	290 (15.3 ± 3.2)		135 (3.9 ± 0.3)	313 (5.0 ± 0.0)	336 (6.0 ± 0.0)	168 (7.1 ± 0.3)	
No. of cases/person-years	35/836.2	39/1182.3	38/1341.6	50/1498.8		31/635.4	48/1624.3	47/1757.0	36/842.3	
Model 1^a^	1.00	0.83 (0.53–1.31)	0.71 (0.45–1.12)	0.85 (0.55–1.31)	0.493	1.00	0.65 (0.41–1.01)	0.57 (0.36–0.90)	0.92 (0.56–1.49)	0.674
Model 2^b^	1.00	0.83 (0.53–1.32)	0.68 (0.43–1.08)	0.72 (0.47–1.12)	0.143	1.00	0.64 (0.40–1.01)	0.50 (0.32–0.78)	0.70 (0.43–1.14)	0.112
Model 3^c^	1.00	0.89 (0.56–1.42)	0.76 (0.47–1.23)	0.81 (0.51–1.29)	0.361	1.00	0.70 (0.44–1.14)	0.62 (0.36–1.07)	0.98 (0.51–1.90)	0.876
2nd nutritional consultation										
N (score means ± SD)	161 (3.1 ± 1.3)	102 (7.0 ± 0.8)	76 (10.1 ± 0.9)	109 (15.1 ± 2.8)		288 (3.8 ± 0.4)	241 (5.0 ± 0.0)	99 (6.0 ± 0.0)	16 (7.4 ± 0.6)	
No. of cases/person-years	21/816.4	18/503.4	19/382.4	16/551.5		41/1484.3	41/1232.8	21/494.2	4/80.9	
Model 1^a^	1.00	1.47 (0.78–2.77)	2.10 (1.13–3.92)	1.25 (0.65–2.42)	0.319	1.00	1.25 (0.81–1.93)	1.64 (0.96–2.78)	2.12 (0.76–5.95)	0.034
Model 2^b^	1.00	1.22 (0.64–2.33)	2.05 (1.09–3.88)	1.01 (0.51–1.99)	0.725	1.00	1.03 (0.66–1.61)	1.14 (0.65–1.97)	1.17 (0.41–3.36)	0.630
Model 3^c^	1.00	1.32 (0.67–2.60)	2.45 (1.23–4.86)	1.19 (0.56–2.56)	0.459	1.00	1.10 (0.68–1.76)	1.38 (0.72–2.62)	1.70 (0.50–5.74)	0.285
3rd nutritional consultation										
N (score means ± SD)	32 (3.3 ± 1.2)	31 (7.1 ± 0.7)	32 (10.1 ± 0.9)	69 (15.3 ± 2.9)		87 (3.7 ± 0.4)	93 (5.0 ± 0.0)	86 (6.0 ± 0.0)	21 (7.0 ± 0.2)	
No. of cases/person-years	4/165.8	3/174.3	8/156.5	13/372.5		12/454.4	21/446.8	12/489.3	6/96.5	
Model 1^a^	1.00	0.71 (0.16–3.23)	2.21 (0.61–7.93)	1.60 (0.51–5.05)	0.286	1.00	1.71 (0.84–3.50)	1.00 (0.44–2.28)	1.99 (0.74–5.36)	0.512
Model 2^b^	1.00	0.72 (0.16–3.25)	1.64 (0.44–6.08)	1.58 (0.49–5.10)	0.293	1.00	1.12 (0.54–2.33)	0.66 (0.29–1.53)	1.34 (0.48–3.76)	0.723
Model 3^c^	1.00	0.73 (0.15–3.44)	1.72 (0.44–6.67)	1.88 (0.49–7.23)	0.215	1.00	1.38 (0.64–3.01)	1.25 (0.45–3.45)	3.48 (0.94–12.88)	0.177

*PG-SGA* patient generated-subjective global assessment

^a^Model 1 was adjusted for age at surgery and sex

^b^Model 2 was adjusted for types of operation (STG or TG), and stages (stage I and II or stage III) in addition to variables in model 1

^c^Model 3 was adjusted for weight loss (kg, continuous), body mass index (kg/m^2^, continuous), digestive symptoms (yes or no), and protein intake relative to requirement (≥ 75% or < 75%) at each nutritional consultation in additional to variables in model 2

In a sensitivity analysis in which we included only patients who completed the 1st, 2nd, and 3rd nutritional consultations, we found that high NUTRISCORE scores at 3 months were associated with poor survival; the HR for ≥ 7 compared to ≤ 4 of NUTRISCORE scores was 3.72 (95% CI: 1.14-12,13) (Table4). Among patients who had all three nutritional assessments, in an analysis of two categories of PG-SGA or NUTRISCORE scores, we found an HR of 2.56 (95% CI: 1.02–6.42) for ≥ 9 vs. ≤ 8 of PG-SGA scores assessed at 3 months after surgery (Table5).

**Table 4:** Hazard ratio (HRs) and 95% confidence intervals (CIs) of total mortality according to PG-SGA and NUTRISCORE among patients who received all three consultations

By time point	PG-SGA					NUTRISCORE				
	**≤ 5**	**6–8**	**9–11**	**≥ 12**	*P* **for trend**	**≤ 4**	**5**	**6**	**≥ 7**	*P* **for trend**
1st nutritional consultation										
N (score means ± SD)	26 (3.2 ± 1.4)	41 (7.2 ± 0.8)	35 (10.1 ± 0.9)	43 (15.5 ± 3.5)		32 (4.0 ± 0.0)	86 (5.0 ± 0.0)	98 (6.0 ± 0.0)	62 (7.1 ± 0.4)	
No. of cases/person-years	8/125.0	9/235.4	7/199.3	10/239.1		13/156.5	17/483.0	16/548.3	16/327.5	
Model 1^a^	1.00	0.60 (0.23–1.57)	0.56 (0.20–1.54)	0.69 (0.27–1.79)	0.571	1.00	0.43 (0.21–0.90)	0.37 (0.18–0.77)	0.59 (0.28–1.25)	0.299
Model 2^b^	1.00	0.58 (0.22–1.52)	0.57 (0.20–1.58)	0.56 (0.21–1.48)	0.324	1.00	0.41 (0.20–0.87)	0.36 (0.17–0.74)	0.46 (0.22–0.97)	0.108
Model 3^c^	1.00	0.59 (0.22–1.60)	0.56 (0.19–1.61)	0.80 (0.28–2.25)	0.752	1.00	0.44 (0.20–0.96)	0.52 (0.22–1.25)	0.86 (0.32–2.31)	0.921
2nd nutritional consultation										
N (score means ± SD)	54 (3.0 ± 1.3)	32 (6.9 ± 0.8)	28 (10.1 ± 0.9)	31 (14.7 ± 2.8)		113 (3.8 ± 0.4)	111 (5.0 ± 0.0)	50 (6.0 ± 0.0)	4 (7.8 ± 1.0)	
No. of cases/person-years	12/302.3	8/157.6	8/150.8	6/175.8		19/609.2	31/585.5	10/272.8	2/17.3	
Model 1^a^	1.00	1.30 (0.52–3.22)	1.40 (0.56–3.47)	0.94 (0.35–2.53)	0.904	1.00	1.76 (0.99–3.12)	1.28 (0.59–2.77)	5.00 (1.05–23.74)	0.119
Model 2^b^	1.00	1.02 (0.38–2.71)	1.77 (0.65–4.79)	0.97 (0.34–2.76)	0.765	1.00	1.44 (0.80–2.60)	1.18 (0.53–2.62)	3.51 (0.71–17.44)	0.279
Model 3^c^	1.00	1.12 (0.39–3.20)	2.12 (0.69–6.55)	1.08 (0.32–3.62)	0.639	1.00	1.79 (0.94–3.39)	1.78 (0.70–4.57)	9.86 (1.46–66.76)	0.057
3rd nutritional consultation										
N (score means ± SD)	26 (3.3 ± 1.1)	25 (7.1 ± 0.7)	29 (10.1 ± 0.9)	65 (15.2 ± 2.9)		85 (3.7 ± 0.4)	91 (5.0 ± 0.0)	81 (6.0 ± 0.0)	21 (7.0 ± 0.2)	
No. of cases/person-years	5/138.0	3/139.2	8/143.2	18/349.3		14/440.3	28/436.6	13/458.2	7/96.5	
Model 1^a^	1.00	0.59 (0.14–2.51)	1.90 (0.59–6.14)	1.65 (0.60–4.55)	0.150	1.00	1.99 (1.04–3.80)	0.94 (0.43–2.07)	2.04 (0.81–5.13)	0.564
Model 2^b^	1.00	0.55 (0.13–2.35)	1.45 (0.44–4.84)	1.69 (0.60–4.70)	0.117	1.00	1.45 (0.75–2.81)	0.69 (0.32–1.52)	1.59 (0.61–4.13)	0.797
Model 3^c^	1.00	0.68 (0.15–3.02)	1.77 (0.50–6.23)	2.47 (0.74–8.28)	0.050	1.00	1.81 (0.90–3.66)	1.24 (0.49–3.16)	3.72 (1.14–12.13)	0.130

*PG-SGA* patient generated-subjective global assessment

^a^Model 1 was adjusted for age at surgery and sex

^b^Model 2 was adjusted for types of operation (STG or TG), and stages (stage I and II or stage III) in addition to variables in model 1

^c^Model 3 was adjusted for weight loss (kg, continuous), body mass index (kg/m^2^, continuous), digestive symptoms (yes or no), and protein intake relative to requirement (≥75% or < 75%) at each nutritional consultation in additional to variables in model 2

**Table 5:** Hazard ratio (HRs) and 95% confidence intervals (CIs) of total mortality according to cut-off points of PG-SGA and NUTRISCORE among patients who received all three consultations

By time point	PG-SGA		NUTRISCORE	
	**≤ 8**	**≥ 9 scores**	**≤ 4 scores**	**≥ 5 scores**
1st nutritional consultation				
N (score means ± SD)	67 (5.6 ± 2.2)	78 (13.1 ± 3.8)	32 (4.0 ± 0.0)	246 (5.9 ± 0.8)
No. of cases/person-years	17/360.4	17/438.4	13/156.5	49/1358.9
Model 1^a^	1.00	0.84 (0.43–1.68)	1.00	0.45 (0.24–0.83)
Model 2^b^	1.00	0.78 (0.39–1.55)	1.00	0.41 (0.22–0.75)
Model 3^c^	1.00	0.91 (0.44–1.88)	1.00	0.48 (0.24–0.98)
2nd nutritional consultation				
N (score means ± SD)	86 (4.4 ± 2.2)	59 (12.6 ± 3.1)	113 (3.8 ± 0.4)	165 (5.4 ± 0.6)
No. of cases/person-years	20/459.8	14/326.5	19/609.2	43/875.6
Model 1^a^	1.00	1.05 (0.52–2.10)	1.00	1.65 (0.96–2.85)
Model 2^b^	1.00	1.28 (0.62–2.64)	1.00	1.39 (0.79–2.44)
Model 3^c^	1.00	1.41 (0.63–3.18)	1.00	1.70 (0.91–3.19)
3rd nutritional consultation				
N (score means ± SD)	51 (5.2 ± 2.1)	94 (13.6 ± 3.4)	85 (3.7 ± 0.4)	193 (5.6 ± 0.7)
No. of cases/person-years	8/277.2	26/492.4	14/440.3	48/991.3
Model 1^a^	1.00	2.16 (0.96–4.86)	1.00	1.68 (0.86–2.91)
Model 2^b^	1.00	2.11 (0.92–4.85)	1.00	1.15 (0.62–2.14)
Model 3^c^	1.00	2.56 (1.02–6.42)	1.00	1.67 (0.84–3.32)

*PG-SGA* patient generated-subjective global assessment

^a^Model 1 was adjusted for age at surgery and sex

^b^Model 2 was adjusted for types of operation (STG or TG), and stages (stage I and II or stage III) in addition to variables in model 1

^c^Model 3 was adjusted for weight loss (kg, continuous), body mass index (kg/m^2^, continuous), digestive symptoms (yes or no), and protein intake relative to requirement (≥ 75% or < 75%) at each nutritional consultation in additional to variables in model 2

We found the tendency of increased risk of mortality with high PG-SGA regardless of sex or stage at the 2nd nutritional consultation, but there were no significant associations for either PG-SGA or NUTRISCORE (Additional file 1: Table S1).

We compared the characteristics of patients according to the timing of nutritional consultations (Additional file 1: Table S2). Mean weight loss was 5.2 ± 2.9 kg at the 1st nutritional consultation, but weight further decreased by 6.3 ± 3.9 kg and 7.7 ± 4.3 kg, respectively, at the 2nd and 3rd nutritional consultations. In addition, the number of meals and eating speed decreased at the 3rd nutritional consultation than at the 1st nutritional consultation. However, the average intake of energy and protein was the lowest at the 1st nutritional consultation (1333.4 ± 391.8 kcal/day, 53.0 ± 19.6 g/day), the highest at the 2nd nutritional consultation (1525.7 ± 413.1 kcal/day, 58.2 ± 20.4 g/day), and then decreased at the 3rd nutritional consultation (1492.5 ± 430.5 kcal/day, 53.9 ± 19.7 g/day). The majority of patients (74.5%) reported experiencing gastrointestinal symptoms such as nausea, decreased appetite, and premature satiety at the 1st nutritional consultation. The proportions of patients who experienced digestive symptoms were 85.7% at the 2nd nutritional consultation and 87.4% at the 3rd nutritional consultation.

When we compared the characteristics between patients who did not participate in the 3rd consultation and those who did, those who participated in the 3rd consultation were younger and had a higher proportion of CCRTx but had similar disease stages (Additional file 1: Table S3).

## Discussion

In this cohort study of gastric cancer patients, we found that high scores of PG-SGA and NUTRISCORE assessed at 2 or 3 months after gastrectomy were associated with poor survival. However, high PG-SGA and NUTRISCORE scores at 1 month after gastrectomy were not associated with poor survival, suggesting that an early evaluation of the PG-SGA and NUTRISCORE right after surgery may not predict prognostic outcomes among gastric cancer patients who underwent surgery. In our study, nutritional assessment at 1 month after surgery was mostly performed before the start of chemotherapy, and assessment at 2 or 3 months after surgery was done during chemotherapy. We found that the risk of mortality in 9–11 scores of PG-SGA was significantly higher, but not that in 12 or more at the second consultation. Whether PG-SGA scores have a non-linear association with mortality risk needs further investigation.

Gastric cancer patients undergoing postoperative chemotherapy often experience chemotherapy-related adverse effects, including nausea/vomiting, anorexia, diarrhea, and stomatitis, leading to reduced food intake, weight loss, and malnutrition [25]. Postoperative gastric stasis, known as delayed gastric emptying, may worsen these symptoms in gastric cancer patients [26, 27]. Therefore, a proper diagnosis of malnutrition is essential to provide adequate nutrition support and intervention for cancer patients [28]. In particular, it emphasizes the importance of nutritional intervention to improve eating after gastrectomy and prevent malnutrition and excessive weight loss [29].

The PG-SGA comprises clinician and patient-centered assessments [20]. The PG-SGA has been recommended by the Oncology Nutrition Dietetic Practice Group of the Academy of Nutrition and Dietetics as the standard for nutrition assessment for cancer patients [21, 28]. A few cohort studies have reported on the prediction capacity of the PG-SGA for survival among gastric cancer patients. A retrospective cohort study of 256 Taiwanese patients with metastatic gastric cancer showed that PG-SGA assessed within one week before chemotherapy was independently associated with survival. In that study, HR (95% CI) for severely malnourished patients (PG-SGA C) compared to patients with PG-SGA A/B was 2.73 (95% CI 1.73–4.29) [30]. Another cohort study of 120 palliative care patients in Brazil, including the patients with gastrointestinal tumors (25.8%, *n* = 31), evaluated the PG-SGA within 24h of hospitalization and found that one score increase in the PG-SGA scores was associated with a 4% increase in mortality [31]. In a Brazilian prospective study of 178 patients with gastric and colorectal cancer, malnourished status assessed by PG-SGA was associated with a 2.9 times higher risk of overall mortality [32]. Those studies included patients with metastatic gastric cancer or palliative care, whereas we included the patients who underwent curative gastrectomy. Our current study of patients who underwent curative gastrectomy also showed a higher risk of mortality among patients with high PG-SGA scores assessed at 2 months after surgery.

The NUTRISCORE, a newly developed cancer-specific nutrition assessment tool, has not been previously evaluated regarding cancer survival. However, a few studies recently reported comparing the NUTRISCORE with other nutritional assessment tools [11, 16, 33]. To the best of our knowledge, no studies have reported the association with survival through multiple nutritional assessments using PG-SGA or NUTRISCORE during the post-gastrectomy follow-up period. The reason why we observed a lower risk of mortality with high NUTRISCORE scores assessed at 1 month after surgery is unclear. However, it is possible that scoring early after surgery may not reflect nutritional status associated with survival, or scoring based on weight loss, appetite, and treatment may not be desired near the start of anticancer therapy. We observed that increase in NUTRSCORE scores at 3 months after surgery was associated with high mortality among gastric patients who underwent curative gastrectomy and received at least 1 cycle of chemotherapy. Our findings may warrant further research.

Regarding the timing of the nutritional assessment in relation to survival, only a few previous studies evaluated nutritional status more than once. Cancer survival studies that used PNI or NRI calculated by a nutrition-related biochemical marker such as albumin and total lymphocyte count (TLC) or weight loss [17, 18] reported that PNI or NRI might be a potential prognostic factor for cancer prognosis. Results of the nutritional evaluation with the NRI after gastrectomy in 760 Japanese patients with stage I-III gastric cancer showed that the overall survival rate was lower among the malnourished group (NRI ≤ 97.5) before the gastrectomy (HR = 1.68; 9% CI: 1.14–2.48), at 1 month (HR = 1.77; 9% CI: 1.22–2.56), at 3 months (HR = 2.18; 9% CI: 1.49–3.21), at 6 months (HR = 1.81; 9% CI: 1.23–2.65), and at 12 months (HR = 2.17; 9% CI: 1.43–3.29) after gastrectomy. Also, malnutrition at 1 month (HR = 1.73, 9% CI: 1.06–2.83) and at 3 months (HR = 1.98, 9% CI: 1.20–3.28) after gastrectomy was significantly associated with poor cause-specific survival [17]. A retrospective study of 1,415 Korean gastric cancer patients who underwent gastrectomy in our hospital found that a decline in PNI scores from preoperative to postoperative 3 months was associated with a 1.53 times higher risk of death, but the PNI scores assessed before surgery or 6 or 12 months after surgery were not associated with risk of death [18].

In the present study, we found that malnutrition at a median of 2 and 3 months after surgery was associated with a poor survival. There was no significant association for the PG-SGA or an inverse association for NUTRISCORE assessed at 1 month. Although the reasons were not clear, nutritional status right after surgery could be affected by gastrointestinal symptoms and dietary intake reduction due to the surgical procedure and acute surgical stress and short-term conditions. Because nutritional status immediately after surgery may reflect surgical stress, it may not sufficiently predict the long-term survival of patients with stage II and III gastric cancer. Further cohort studies are needed on whether there is a difference in the association between malnutrition and cancer survival according to the nutritional evaluation timing. Also, it is possible that it takes time to detect progressive malnutrition status associated with survival given continued weight loss [34].

Our study has several limitations. First, because the 2nd and 3rd nutritional consultations were conducted only when the patient wanted, we did not have all the initial group of patients at the 2nd and 3rd consultations compared to the 1st consultation. However, we found similar clinical characteristics, including disease progression and stage, between patients who participated in the 2nd and 3rd consultations and those who did not. Also, when we included only patients who received all three nutritional consultations, we observed an increase in mortality with high scores. Second, we cannot rule out the possibility of potential unknown or residual confounding factors. Third, our results may not be generalizable to all gastric cancer patients; nevertheless, given our study center receives referral patients from all over the country, the generalizability may not be an issue. The strength of our study is examining the association between mortality and malnutrition evaluated according to the timing of at least one postoperative nutritional consultation in the same group of gastric cancer patients. Our study provides insight that efforts should be made to improve survival by intensive nutritional intervention for malnutrition patients during initial 2–3 months of chemotherapy after gastrectomy.

## Conclusion

The present study showed that malnutrition assessed by the PG-SGA and NUTRISCORE at 2–3 months, but not at 1 month, after surgery was related to the prognosis among gastric cancer patients. Patients with high PG-SGA and NUTRISCORE scores at 2–3 months had a lower survival rate. Our study suggests that malnutrition diagnosis through PG-SGA or NUTRISCORE evaluation and nutritional intervention at 2–3 months during chemotherapy after surgery may be an appropriate time to improve the prognosis in gastric cancer patients.

## Electronic supplementary material

Below is the link to the electronic supplementary material.

Supplementary Material 1. Table S1 Hazard ratio (HRs) and 95% confidence intervals (CIs) for all-cause mortality according to PG-SGA and NUTRISCORE scores by subgroups of gastric cancer patients at 2nd nutritional consultation after surgery. Table S2 General characteristics of gastric cancer patients at each time point of postoperative nutritional consultation in patients with NUTRISCORE scores. Table S3 Baseline characteristics according to the participation of the 3rd consultation.

## Data Availability

The data that support the findings of this study are available from Samsung Medical Center but restrictions apply to the availability of these data and so are not publicly available. Data are however available from the authors upon reasonable request and with permission of Samsung Medical Center.

## Abbreviations

PG-SGA: patient generated-subjective global assessment
STG: subtotal gastrectomy
TG: total gastrectomy
EGC: early gastric cancer
AGC: advanced gastric cancer
CTx: chemotherapy
CCRTx: combined chemoradiotherapy
TLC: total lymphocyte count
