# Supplementary material for: Associations of patient-generated subjective global assessment (PG-SGA) and NUTRISCORE with survival in gastric cancer patients: timing matters, a retrospective cohort study
Source: BMC Gastroenterol. 2022 Nov 17;22:468. doi: 10.1186/s12876-022-02515-3 (PMC9673437; doi:10.1186/s12876-022-02515-3)
Supplement: Supplementary file 1 — Supplementary Material 1. Table S1 Hazard ratio (HRs) and 95% confidence intervals (CIs) for all-cause mortality according to PG-SGA and NUTRISCORE scores by subgroups of gastric cancer patients at 2nd nutritional consultation after surgery. Table S2 General characteristics of gastric cancer patients at each time point of postoperative nutritional consultation in patients with NUTRISCORE scores. Table S3 Baseline characteristics according to the participation of the 3rd consultation. [file 12876_2022_2515_MOESM1_ESM.docx]

**Table S1** Hazard ratio (HRs) and 95% confidence intervals (CIs) for all-cause mortality according to PG-SGA and NUTRISCORE scores by subgroups of gastric cancer patients at 2^nd^ nutritional consultation after surgery

| **Characteristic** | **PG-SGA** | | **NUTRISCORE** | |
| --- | --- | --- | --- | --- |
|  | **≤ 8 scores** | **≥ 9 scores** | **≤ 4 scores** | **≥ 5 scores** |
| Men |  |  |  |  |
| N (score means ± SD) | 177 (4.3 ± 2.2) | 107 (12.5 ± 3.1) | 199 (3.7 ± 0.4) | 209 (5.4 ± 0.6) |
| No. of cases/person-years | 32/893.9 | 30/517.2 | 43/1010.6 | 50/1050.0 |
| Model^a^ | 1.00 | 1.61 (0.91-2.84) | 1.00 | 0.94 (0.58-1.53) |
| Women |  |  |  |  |
| N (score means ± SD) | 86 (5.2 ± 2.3) | 78 (13.8 ± 3.5) | 89 (3.9 ± 0.4) | 147 (5.4 ± 0.7) |
| No. of cases/person-years | 14/426.0 | 15/416.7 | 14/473.7 | 32/757.8 |
| Model^a^ | 1.00 | 1.60 (0.69-3.69) | 1.00 | 1.39 (0.64-3.05) |
| Stage I and II of gastric cancer |  |  |  |  |
| N (score means ± SD) | 138 (4.3 ± 2.3) | 99 (12.9 ± 3.2) | 174 (3.8 ± 0.4) | 185 (5.3 ± 0.6) |
| No cases/person-years | 7/760.4 | 9/569.5 | 13/983.4 | 20/1043.9 |
| Model^b^ | 1.00 | 1.68 (0.59-4.81) | 1.00 | 1.89 (0.82-4.36) |
| Stage 3 of gastric cancer |  |  |  |  |
| N (score means ± SD) | 125 (4.9 ± 2.2) | 86 (13.2 ± 3.5) | 114 (3.8 ± 0.4) | 171 (5.5 ± 0.7) |
| No cases/person-years | 39/559.4 | 36/364.4 | 44/501.0 | 62/763.9 |
| Model^b^ | 1.00 | 1.38 (0.83-2.29) | 1.00 | 0.84 (0.53-1.33) |

*PG-SGA* patient generated-subjective global assessment

^a^Model was adjusted for age at surgery, types of operation (STG or TG), stages (stage I & II or stage III), weight loss (kg, continuous), body mass index (kg/m^2^, continuous), digestive symptoms (yes or no), and protein intake relative to requirement (≥75% or <75%).

^b^Model was adjusted for age at surgery, sex, types of operation (STG or TG), weight loss (kg, continuous), body mass index (kg/m^2^, continuous), and protein intake relative to requirement (≥ 75% or < 75%).

**Table S2** General characteristics of gastric cancer patients at each time point of postoperative nutrition consultation in patients with NUTRISCORE scores

| **Characteristics^a^** | **1st consultation** | **2nd consultation** | **3rd consultation** |
| --- | --- | --- | --- |
| N | 952 | 644 | 287 |
| Age at surgery (years) | 55.3 ± 11.5 | 53.8 ± 11.0 | 52.8 ± 10.8 |
| Sex |  |  |  |
| Men | 622 (65.3) | 408 (63.4) | 183 (63.8) |
| Women | 330 (34.7) | 236 (36.7) | 104 (36.2) |
| Body Mass Index (kg/m^2^) | 21.8 ± 2.7 | 21.4 ± 2.6 | 21.0 ± 2.6 |
| Weight loss (kg) | 5.2 ± 2.9 | 6.3 ± 3.9 | 7.7 ± 4.3 |
| TNM stage |  |  |  |
| Stage I and II | 528 (55.5) | 359 (55.8) | 160 (55.8) |
| Stage III | 424 (44.5) | 285 (44.3) | 127 (44.3) |
| Type of operation |  |  |  |
| STG | 618 (64.9) | 418 (64.9) | 191 (66.6) |
| TG | 334 (35.1) | 226 (35.1) | 96 (33.5) |
| Disease progression |  |  |  |
| EGC | 149 (15.7) | 102 (15.8) | 43 (15.0) |
| AGC | 803 (84.4) | 542 (84.2) | 244 (85.0) |
| Anticancer treatment |  |  |  |
| CTx only | 498 (52.3) | 308 (47.8) | 92 (32.1) |
| CCRTx | 454 (47.7) | 336 (52.2) | 195 (67.9) |
| Postoperative hospital stay (days) | 11.4 ± 4.0 | 11.2 ± 3.1 | 11.0 ± 2.5 |
| Energy intake (kcal/day) | 1333.4 ± 391.8 | 1525.7 ± 413.1 | 1492.5 ± 430.5 |
| Energy intake relative to requirement (%) | 71.0 ± 19.0 | 81.3 ± 19.6 | 79.8 ± 21.0 |
| Protein intake (g/day) | 53.0 ± 19.6^b^ | 58.2 ± 20.4^c^ | 53.9 ± 19.7^d^ |
| Protein intake relative to requirement (%) | 71.7 ± 25.1^b^ | 78.6 ± 26.0^c^ | 72.9 ± 25.5^d^ |
| Number of meals (times/day) | 3.5 ± 1.0^b^ | 3.1 ± 0.4^c^ | 3.0 ± 0.3^d^ |
| Number of snacks (times/day) | 2.7 ± 1.0^b^ | 2.6 ± 0.8^c^ | 2.6 ± 0.7^d^ |
| Eating speed (minutes) | 20.3 ± 6.4^b^ | 19.8 ± 7.0^c^ | 18.4 ± 6.3^d^ |
| Postprandial digestive problems (yes, %) | 709 (74.5) | 547 (85.7)^c^ | 250 (87.4)^d^ |

*STG* subtotal gastrectomy, *TG* total gastrectomy, *EGC* early gastric cancer, *AGC* advanced gastric cancer, *CTx* chemotherapy, *CCRTx* combined chemoradiotherapy

^a^Values were presented as mean ± standard deviation for continuous variables or number (%) for categorical variables.

^b^There were fewer patients due to a lack of information: protein intake and protein intake relative to requirement, 894; the number of meals, 951; the number of snacks, 931; eating speed, 650.

^c^There were fewer patients due to a lack of information: protein intake and protein intake relative to requirement, 619; the number of meals, 642; the number of snacks, 631; eating speed, 351; postprandial digestive problems, 638.

^d^There were fewer patients due to a lack of information: protein intake and protein intake relative to requirement, 273; the number of meals, 280; the number of snacks, 268; eating speed, 154; postprandial digestive problems, 286.

**Table S3** Baseline characteristics according to the participation of the 3^rd^ consultation

| **Characteristics^a^** | **Total** | **3^rd^ consultation** | | ***P* value** |
| --- | --- | --- | --- | --- |
|  |  | **No** | **Yes** |  |
| N (%) | 952 | 665 (69.9) | 287 (30.2) |  |
| Person-years | 4858.98 | 3285.4 | 1573.6 |  |
| Age at surgery (years) | 55.3 ± 11.5 | 56.4 ± 11.6 | 52.8 ± 10.8 | <0.001 |
| Sex |  |  |  |  |
| Men | 622 (65.3) | 439 (66.0) | 183 (63.8) | 0.503 |
| Women | 330 (34.7) | 226 (34.0) | 104 (36.2) |  |
| Body Mass Index (kg/m^2^) | 21.8 ± 2.7 | 21.7 ± 2.7 | 22.0 ± 2.7 | 0.073 |
| Weight loss (kg) | 5.2 ± 2.9 | 5.3 ± 2.8 | 5.0 ± 3.3 | 0.308 |
| TNM stage |  |  |  |  |
| Stage I and II | 528 (55.5) | 368 (55.3) | 160 (55.8) | 0.907 |
| Stage III | 424 (44.5) | 297 (44.7) | 127 (44.3) |  |
| Type of operation |  |  |  |  |
| STG | 618 (64.9) | 427 (64.2) | 191 (66.6) | 0.488 |
| TG | 334 (35.1) | 238 (35.8) | 96 (33.5) |  |
| Disease progression |  |  |  |  |
| EGC | 149 (15.7) | 106 (15.9) | 43 (15.0) | 0.709 |
| AGC | 803 (84.4) | 559 (84.1) | 244 (85.0) |  |
| Anticancer treatment |  |  |  |  |
| CTx only | 498 (52.3) | 406 (61.1) | 92 (32.1) | <0.001 |
| CCRTx | 454 (47.7) | 259 (39.0) | 195 (67.9) |  |
| Postoperative hospital stay (days) | 11.4 ± 4.0 | 11.6 ± 4.5 | 11.0 ± 2.5 | 0.088 |
| Energy intake (kcal/day) | 1333.4 ± 391.8 | 1318.7 ± 403.3 | 1367.5 ± 362.0 | 0.042 |
| Energy intake relative to requirement (%) | 71.0 ± 19.0 | 70.4 ± 19.8 | 72.4 ± 17.2 | 0.115 |
| Protein intake (g/day)^b^ | 53.0 ± 19.6 | 51.6 ± 20.1 | 56.0 ± 18.2 | <0.001 |
| Protein intake relative to requirement (%)^b^ | 71.7 ± 25.1 | 69.9 ± 26.0 | 75.6 ± 22.5 | <0.001 |
| Number of meals (times/day)^b^ | 3.5 ± 1.0 | 3.5 ± 1.0 | 3.5 ± 0.9 | 0.391 |
| Number of snacks (times/day)^b^ | 2.7 ± 1.0 | 2.7 ± 0.7 | 2.9 ± 1.5 | <0.001 |
| Eating speed (minutes)^b^ | 20.3 ± 6.4 | 19.7 ± 6.2 | 21.3 ± 6.6 | <0.001 |
| Postprandial digestive problems (yes, %) | 709 (74.5) | 480 (72.2) | 229 (79.8) | 0.014 |

*STG* subtotal gastrectomy, *TG* total gastrectomy, *EGC* Early gastric cancer, *AGC* advanced gastric cancer, *CTx*, chemotherapy, *CCRTx* combined chemoradiotherapy

^a^Values were presented as mean ± standard deviation for continuous variables or number (%) for categorical variables.

^b^There were fewer patients due to a lack of information: protein intake and protein intake relative to requirement, 894; the number of meals, 951; the number of snacks, 931; eating speed, 650.
